# Physical Activity, Screen-Based Sedentary Behavior and Physical Fitness in Chinese Adolescents: A Cross-Sectional Study

**DOI:** 10.3389/fped.2021.722079

**Published:** 2021-10-05

**Authors:** Xiaosheng Dong, Lijie Ding, Rui Zhang, Meng Ding, Baozhen Wang, Xiangren Yi

**Affiliations:** ^1^College of Physical Education, Shandong University, Jinan, China; ^2^Department of Health Management, Shandong Sports University, Jinan, China; ^3^College of Physical Education, Shandong Normal University, Jinan, China; ^4^Department of Toxicology and Nutrition, School of Public Health, Cheeloo College of Medicine, Shandong University, Jinan, China

**Keywords:** physical activity, screen-based sedentary behavior, physical fitness, adolescents, China

## Abstract

**Purpose:** The aim of this study is to explore the relationship between screen-based sedentary behavior, physical activity and physical fitness among Chinese adolescents.

**Methods:** This study randomly selected adolescents from 10 administrative districts in Shandong, China. The data gathering tools for demographic and other characteristics (gender, age, body mass index and socioeconomic status), PA (PAQ-A) and screen-based sedentary behavior (YRBSS) and physical fitness (NSPFH 2014) were utilized in this study. Statistical analysis was performed by *T*-test, chi-square test and multiple linear regression.

**Results:** 10,002 adolescents (14.39 years ± 1.79) participated in the study. The results demonstrated that BMI and high TV viewing time had a significant negative correlation with physical fitness, but there was no association between the amount of time spent playing computer/video games and physical fitness among adolescents. High SES and physical activity in leisure time five or more times per week were significantly associated with most dimensions of physical fitness.

**Conclusions:** the results suggest that we not only need to focus on adolescent risk behavior associated with low socioeconomic status and obesity, but also enforce physical activity and reduce sedentary television-watching behavior, which will be crucial pathways and strategies to improve the physical fitness of Chinese adolescents.

## Introduction

Physical fitness has become a crucial prognosticator of adolescent health (1) and significantly associated with gauges of health such as cardiovascular health (2), cognitive capability and psychological well-being (3). Relevant studies indicate that physical inactivity is not only an independent risk factor for chronic diseases such as hypertension (4), heart disease (5) and 2 diabetes mellitus (6), but also has a serious negative impact on physical fitness, which leads to massive social issues (7). Globally, 80% of adolescents are lacking in physical activity; “low physical activity-high sedentary time” has become a widely-used descriptor of current physical inactivity among adolescents (8–10). The latest WHO guidelines recommend that children and adolescents should engage in an average of 60 min per day of moderate to high intensity exercise (mainly aerobic exercise), and limit sedentary time, especially screen time (11).

A current study from 39 countries finds that only 23 and 19% of children aged 11–13 years old, respectively, meets the recommended levels of physical activity, and that contemporary adolescents engage in physical activity with less frequently and for shorter durations than their parents (12). In addition, since our society has launched into the digital age, smart devices such as television, computers and video games have become so accessible for children and adolescents as to become an integral and habitual part of their lives (13). The result is the frequency and duration of their screen time exceeds recommended limits (14, 15).

Screen-based sedentary behavior has ascended as an independent factor affecting the physical fitness of children and adolescents (16). At present, the declining tendency of physical fitness level in adolescents has gradually become a severe problem that we are faced with in China (17, 18). Recent studies find Chinese adolescents have the dual challenge of more daily homework and screen time, which greatly reduces leisure-time physical activity and increases a sedentary lifestyle (19, 20). The latest prevalence estimates are 35 and 37% of children and adolescents in China reported spending more than 2 h a day with electronic screens (i.e., TV, computers, smartphones, digital tablets and video games) from 2016 to 2017 (21).

Studies point out the relationship between the duration of screen use and physical fitness in adolescents, that is, the longer the duration of television-watching per day, the higher the risk of physical fitness decline (22–24). Sedentary behavior is associated with lower muscle strength and endurance (25) and lower physical fitness levels (26), which results in health hazards of reduced cardiorespiratory fitness, muscle strength and endurance, increasing adiposity and affects mental health, sleep, social behavior and quality of life (27, 28). The research demonstrates that children with high screen exposure have a negative relationship with sports development and are more likely to have gross motor development problems (29). The poor executive function and low level of motor development increases musculoskeletal risk (30), as well as reduced cardiopulmonary function, muscle strength and endurance (27, 28). Research suggests that children and adolescents who engage in 60 min or more of moderate to vigorous physical activity per day benefit greatly across multiple areas of physical fitness, with the resulting positive effects lasting into their lifetime (31). However, excessive screen time is likely to lead to reducing physical activity (32). Poor behavioral habits developed during childhood and adolescence may extend into adulthood and affect the construction of a healthy lifestyle (33). The health hazards of screen-based sedentary behavior are a long-term, cumulative process that may influence physical fitness in adulthood (34). However, the impact of screen behavior as an independent hazard feature to the health of children and adolescents has converted into an important public health issue (35, 36).

Fostering healthy lifestyles, improving physical activity levels and reducing screen behavior of children and adolescents are urgently needed to promote physical fitness in China, and are also imperative to accomplish the strategic target of Healthy China (37, 38). Although relevant studies have investigated the association between physical activity, sedentary behavior and physical fitness of adolescents (23, 39, 40), a larger population should be studied in order to verify the effects of these three variables among Chinese adolescents. The purpose of this study is to explore the relationship between screen-based sedentary behavior, physical activity and physical fitness among Chinese adolescents through a large of population and indentify demographic factors affecting physical fitness, such as age, BMI, and SES. We hypothesized that high physical activity and low screen-based sedentary behavior are associated with the better physical fitness of Chinese adolescents, and demographic factors (e.g., age, BMI, and SES) affecting physical fitness. The knowledge gained through this study may facilitate the development of physical fitness promotion policies and programs for Chinese adolescents.

## Materials and Methods

### Design, Setting, and Participants

A cross-sectional study was conducted by students recruited from 100 schools of 10 districts in Shandong Province, China, in the 2017–2018 semester. According to the specific geographical, demographic and socio-economic levels of the districts (41), 30 high school and 70 middle schools were randomly selected from 10 administrative districts. Three high schools and 7 middle schools were randomly selected in each district, with at least 100 students in each grade and over 300 students in each school. After screening, a total of 10,002 students (14.39 ± 1.79 years; BMI_mean_ = 20.36) finally completed all the questionnaires and physical fitness tests of the research institute, of which 49.54% were girls (*n* = 4,955; BMI_mean_ = 20.21) and 50.46% were boys (*n* = 5,047 BMI_mean_ = 20.50).

A total of 90 evaluators were recruited from physical education (PE) teachers working in middle and high schools who had previous experience in evaluating youth fitness and who had operated National Student Fitness Test program. In order to ensure the standardization of the test and decrease the error of the test, all PE teachers completed two training for test procedures and other matters needing attention. The trained investigators employed the standardized guides to organized students to measure physical fitness and guided students to answer online questionnaires. It was well-noted by all participants that all data was collected voluntarily, anonymously and confidentially, reserved on a password-protected website and accessible only to direct researchers. Both parents and students completed informed consent forms before beginning this survey. This study has been approved by the Ethics Committee of Shandong University.

### Study Variables

#### Demographic and Other Characteristics

Adolescents reported basic information, and socioeconomic status (SES) and body mass index (BMI) data were collected as well-because they are associated with dependent variables to have a confusing effect on the statistical results. The SES of guardians was investigated from the aspects of educational background and occupational status (42). An individual's SES score was calculated by multiplying an occupation scale value by a weight of 5 and education scale value by a weight of 3. Educational scale value ranged from 3 to 18 while occupational scale value ranged from 5 to 30. The total SES index ranged from 8 to 48 and was categorized as high (35–48), moderate (22–34), and low (8–21) (43). The validity and reliability of this instrument were endorsed by Cirino et al. (42). Body mass index (BMI) was applied to assess adolescents' weight status. A digital electronic scale (HW-VB900, Lejia, China) was used to measure the weight and height of barefoot students wearing light clothing with an accuracy of 0.1 kg. The calculation formula is weight (kg)/height 2 (m2).

#### Physical Activity

Physical activity was assessed using the Physical Activity Questionnaire for Adolescents (PAQ-A). This scale is a revised version of the Physical Activity Questionnaire for Older Children (PAQ-C), which aims to assess the level of physical activity of adolescents (44). Its effectiveness and reliability have been verified among Chinese adolescents (45). This questionnaire mainly asks adolescents what they did in most of their free time in the past 7 days. The physical activity level is scored on a 5-point scale (1–5), with a higher score indicating a higher PA level. It can be divided into low PA level (1–1.9 points) and high PA level (2–5 points) (46). Reliability of the questionnaire was analyzed by Cronbach's alpha (α = 0.821). Those question asked: Which of the following best describes your performance in the past week? “I spend almost all my free time doing activities that have nothing to do with physical activity”; “I sometimes (once or twice in the last week) do some physical activity in my free time (e.g., exercise, running, swimming, cycling, aerobics, etc.)”; “I often (3–4 times in the last week) do some physical activity in my free time”; “I often (5–6 times in the last week) do some sports in my free time”; “I do some physical activity in my free time very often (7 times or more in the last week).”

#### Screen-Based Sedentary Behavior

Adolescents' sedentary behavior was assessed by two YRBSS questions (47): “During the semester, on Monday through Friday, how many hours of TV did you watch on an average day? On an average day, Monday to Friday of this semester, how many hours per day do you spend playing video games or using the computer for non-study activities (including time spent on QQ, WeChat, iPad or other social software such as texting or other social software)?” Each question has seven response options ranging from I don't watch TV/play video games or use the computer for non-academic things when I'm at school to ≥5 h. In the analysis, according to this classification, the time spent on sedentary behavior was recoded as (I) <3 h and (ii) ≥3 h (48).

#### Physical Fitness

National Student Physical Fitness and Health 2014 (NSPFH 2014) (49) was used to evaluate proficiency in the following aspects of physical fitness: 50-m sprint, sit and reach, standing long jump, bent-leg sit-ups for girls, pull-ups for boys, 1,000-m run for boys, and 800-m run for girls. These test items are reliable and effective tools to measure the physique of teenagers in China.

50-m sprint: We took the 50-meter sprint test to assess the students' speed and explosive power. When the subjects heard the “go” command, they began a 50-meter run. They ran the whole course as fast as they could. Time was recorded in minutes and seconds.

Sit and reach: In order to evaluate low back flexibility, sit and reach activity was measured. Every barefoot subject sat on the instrument and gradually extended his or her knees forward. The test was recorded twice, and the better score was retained.

Standing long jump: To measure lower-limb explosive strength, standing long jump was introduced. Every subject was asked to stand at the starting line and jump forward as far as possible. It was measured in meters from the starting line to the heel of the closest foot. The test was recorded three times, and the better score was retained.

1,000/800-m run: Every student stood at the scratch line and was asked to complete the 800 or 1,000 meters as fast as possible. Time was recorded in minutes and seconds. All the girls ran 800 meters and the boys ran 1,000 meters.

Pull-ups: The upper body muscular strength was tested by pull-ups. The test was scored on the number of pull-ups. The subject jumped up and pulled on the railing with both hands. After standing still, subjects pulled up with both arms together. All the male students were tested.

Bent-leg sit-ups: Every subject was asked to lie on a mat with knees bent 90 degrees, the upper body raised and elbows touching knees. The number of bent-leg sit-ups finished in 1 min was recorded. All the female students were tested.

#### Statistical Analysis

All statistical analyses were performed using IBM SPSS Statistics for Windows (Statistics 25, IBM Corporation, Chicago, USA). Data were tested for normality with the Shapiro–Wilk test. The *t*-test and the chi-square test were used for all variables in terms of gender. Continuous variables were represented by the mean and standard deviation (mean ± standard deviation), while classified variables were represented by a number (*n*) and percentage (%). Linear regression was used to analyze the relationship between age, socioeconomic status, sedentary screen behavior, physical activity and physical fitness. In addition to screen-related sedentary behavior (regarding physical activity) and physical activity (regarding screen-based sedentary behavior), all models were adjusted for age, BMI, and SES. Results in all models were expressed as a non-standard coefficient (β) with a 95% confidence interval (95% CI). *P* ≤ 0.05 was statistically significant.

## Results

A total of 10,002 adolescents in this study were chosen in the final statistical analysis, of which 49.54% were girls and 50.46% were boys. Descriptive statistical analysis based on gender (Table 1) showed that the mean age, BMI, 50-m sprint, standing long jump and sit and reach were 14.39 years, 20.36, 8.84 s, 184.09 cm and 11.16 cm, respectively, with significant differences existing between boys and girls. The average endurance for boys (1,000 m) and girls (800 m) was 4.52 s and 4.06 s. The average number of pull-ups for boys and bent-leg sit-ups for girls was 5.35 and 30.35 respectively.

**Table 1:** Characteristics of participants.

**Variables**	**All (*n* = 10,002)**	**Boys (*n* = 5,047)**	**Girls (*n* = 4,955)**	***p*-value**
Age (years)*	14.39 (1.79)	14.32 (1.78)	14.45 (1.80)	0.00
BMI*	20.36 (4.06)	20.50 (3.87)	20.21 (4.25)	0.00
Overweightness/obesity (%)	18.82	19.24	18.39	0.00
50-m sprint (sec)*	8.84 (1.58)	8.26 (1.63)	9.43 (1.28)	0.00
Standing long jump (cm)*	184.09 (33.59)	202.67 (32.49)	165.17 (22.20)	0.00
Sit and reach (cm)*	11.16 (8.62)	9.68 (9.23)	12.68 (7.66)	0.00
1,000-m run (min)		4.52 (0.97)		
800-m run (min)			4.06 (0.68)	
Pull-ups (reps)		5.35 (6.40)		
Bent-leg sit-ups (reps)			30.35 (10.45)	
Screen-based sedentary behavior				
Television viewing, *n* (%)*				0.00
<3 h	9,048 (90.46)	4,507 (89.30)	4,541 (91.64)	
≥3 h	954 (9.54)	540 (10.70)	414 (8.36)	
Computer/videogame use, *n* (%)*				0.00
<3 h	8,826 (88.24)	4,286 (84.92)	4,540 (91.62)	
≥3 h	1,176 (11.76)	761 (15.8)	415 (8.38)	
Physical activity category, *n* (%)*				0.00
Active	5,964 (59.63)	3,212 (63.64)	2,752 (55.54)	
Inactive	4,038 (40.37)	1,835 (36.36)	2,203 (44.46)	
Regular exercise, *n* (%)*				0.00
<1 time pw	1,961 (19.61)	923 (18.29)	1,038 (20.95)	
1–2 times pw	4,104 (41.03)	1,912 (37.88)	2,192 (44.24)	
3–4 times pw	2,282 (22.82)	1,219 (24.15)	1,063 (21.45)	
≥5 times pw	1,655 (16.55)	993(19.68)	662 (13.36)	
SES, *n* (%)				0.32
High	2,696 (26.96)	1,392 (27.58)	1,304 (26.32)	
Moderate	3,057 (30.56)	1,540 (30.51)	1,517 (30.62)	
Low	4,249 (42.48)	2,115 (41.91)	2,134 (42.06)	

Data were described as n (%) or mean ± SD; BMI, Body Mass Index; SES, socioeconomic status; Screen-based SB, Screen-based sedentary behavior; TV, Television viewing; C/V use, Computer/videogame use; pw: per week. *Significant difference between male and female, p < 0.05.

9.54% and 11.76% of adolescents surveyed watched TV and play computer/video games more than 3 h daily, respectively. The average of 40.3% of adolescents had insufficient physical activity. The results showed the exercise frequency of adolescents as follows: 19.61% exercise 0 times/week, 41.03% 1–2 times/week, 22.82%, 3–4 times/week, 16.55% ≥5 times/week. Significant differences were shown between boys and girls in their screen-based sedentary behavior, physical activity, and frequency of physical activity in leisure time; however no significant differences in SES.

As shown in Table 2, the relationship between physical activity and screen-based sedentary behavior and demographic factors and physical fitness of adolescents was analyzed through a multiple linear regression model. Comparing participants on the variable of TV viewing time, the high TV viewing time had a significant impact on physical fitness of 50-m sprint (β:0.452; 95% CI:0.282–0.621), standing long jump (β: −4.562; 95% CI: −7.469 to −1.656) and 1,000-m run (β: 0.107; 95% CI: 0.013–0.201) in boys and 50-m sprint (β: 0.537; 95% CI: 0.388–0.686), sit and reach (β: −1.173; 95% CI: −2.072 to −0.274), 800-m run (β: 0.149; 95% CI: 0.072–0.227) and bent-leg sit-ups (β: −1.383; 95% CI: −2.597 to −0.169) in girls. In addition, there was no association between the amount of time spent playing computer/video games and physical fitness among adolescents. Compared with the the physically inactive (as a reference), those with a high level of physical activity were significantly positively associated with 1,000-m run (β: −0.082; 95% CI: −0.142 to −0.023) and pull-ups (β: 0.466; 95% CI: 0.039–0.894) in boys and standing long jump (β: 1.504; 95% CI: 0.078–2.93) and bent-leg sit-ups (β: 1.07; 95% CI: 0.378–1.762) in girls. Compared with physical activity <1 time per week, the 1–2 times per week was positively associated with the standing long jump (β: 3.461; 95% CI: 1.278–5.645) and 1,000-m run (β: −0.16; 95% CI: −0.23 to −0.089) in boys and 800-m run (β: −0.054; 95% CI: −0.105 to −0.002) in girls. The 3–4 times per week was associated with the standing long jump (β: 3.284; 95% CI: 0.702–5.866) and 1,000-m run-0.131(−0.215 to −0.047) in boys and 50-m sprint (β: −0.131; 95% CI: −0.255 to −0.007) and 800-m run (β: −0.076; 95% CI: −0.14 to −0.011) in girls. Moreover, more than 5 times per week was significantly positively associated with most dimensions of physical fitness (except girls' bent-leg sit-ups).

**Table 2:** Multivariable General Linear Models Evaluating the Association of Physical Activity and Screen-based Sedentary Behavior and Demographic Factors and Physical Fitness.

**Physical fitness test**	**Demographic characteristics**	**Boys (*****n*** **=** **5,047)** **β** **(95% CI)**		**Girls (*****n*** **=** **4,955)** **β** **(95% CI)**	
50-m sprint (s)	BMI	0.039 (0.028, 0.028)**	*F* = 38.500 R^2^ = 0.069	0.019 (0.011, 0.028)**	*F* = 13.617 R^2^ = 0.025
	SES (moderate vs. low)	−0.069 (−0.173, 0.035)		−0.081 (−0.165 to 0.002)	
	SES (high vs. low)	−0.061 (−0.168 to 0.046)		−0.232 (−0.32 to −0.145)**	
	Screen-based SB				
	TV (≥3 h vs. <3 h)	0.452 (0.282, 0.621)**		0.537 (0.388, 0.686)**	
	C/V use (≥3 h vs. <3 h)	−0.096 (−0.242 to 0.05)		−0.059 (−0.207 to 0.09)	
	Physical activity				
	Physical activity (Active vs. Inactive)	−0.066 (−0.173 to 0.04)		−0.015 (−0.1 to 0.07)	
	Regular exercise (1–2 times pw vs. <1 time pw)	−0.11 (−0.237 to 0.017)		−0.098 (−0.196 to 0.001)	
	Regular exercise (3–4 times pw vs. <1 time pw)	−0.096 (−0.247 to 0.054)		−0.131 (−0.255 to −0.007)*	
	Regular exercise (≥5 times pw vs. <1 time pw)	−0.178 (−0.338 to −0.019)*		−0.215 (−0.355 to −0.074)**	
Standing long jump (cm) Sit and reach (cm)	BMI	−1.239 (−1.434 to −1.043)**	*F* = 226.410 R^2^ = 0.309	−0.312 (−0.452 to −0.172)**	*F* = 49.639 R^2^ = 0.090
	SES (moderate vs. low)	2.591 (0.807, 4.375)**		1.453 (0.054, 2.853)*	
	SES (high vs. low)	3.007 (1.168, 4.845)**		2.139 (0.669, 3.609)**	
	Screen-based SB				
	TV (≥3 h vs. <3 h)	−4.562 (−7.469 to −1.656)**		−1.157 (−3.658, 1.344)	
	C/V use (≥3 h vs. <3 h)	1.106 (−1.406 to 3.618)		0.302 (−2.189 to 2.794)	
	Physical activity				
	Physical activity (Active vs. Inactive)	1.475 (−0.353 to 3.302)		1.504 (0.078, 2.93)*	
	Regular exercise (1–2 times pw vs. <1 time pw)	3.461 (1.278, 5.645)**		0.954 (−0.699 to 2.607)	
	Regular exercise (3–4 times pw vs. <1 time pw)	3.284 (0.702, 5.866)*		0.124 (−1.961 to 2.209)	
	Regular exercise (≥5 times pw vs. <1 time pw)	3.782 (1.046, 6.517)**		2.737 (0.386, 5.089)*	
	BMI	−0.101 (−0.168 to −0.035)**	*F* = 4.881 R^2^ = 0.008	−0.008 (−0.058 to 0.042)	*F* = 7.099 R^2^ = 0.012
	SES (moderate vs. low)	0.275 (−0.334 to 0.883)		−0.32 (−0.823 to 0.183)	
	SES (high vs. low)	0.211 (−0.416, 0.839)		0.142 (−0.387 to 0.67)	
	Screen-based SB				
	TV (≥3 h vs. <3 h)	0.059 (−0.932 to 1.05)		−1.173 (−2.072 to −0.274)*	
	C/V use (≥3 h vs. <3 h)	−0.514 (−1.371 to 0.342)		0.654 (−0.241 to 1.55)	
	Physical activity				
	Physical activity (Active vs. Inactive)	−0.39 (−1.013 to 0.233)		0.128 (−0.385 to 0.64)	
	Regular exercise (1–2 times pw vs. <1 time pw)	0.013 (−0.732 to 0.757)		0.33 (−0.264 to 0.924)	
	Regular exercise (3–4 times pw vs. <1 time pw)	−0.03 (−0.91 to 0.851)		0.429 (−0.321 to 1.178)	
	Regular exercise (≥5 times pw vs. <1 time pw)	0.964 (0.031, 1.897)*		0.987 (0.142, 1.833)*	
1,000-m run (min)	BMI	0.052 (0.046, 0.058)	*F* = 122.893 R^2^ = 0.195		
	SES (moderate vs. low)	−0.072 (−0.13 to −0.014)*			
	SES (high vs. low)	−0.099 (−0.158 to −0.039)**			
	Screen-based SB				
	TV (≥3 h vs. <3 h)	0.107 (0.013, 0.201)*			
	C/V use (≥3 hvs. <3 h)	−0.032 (−0.113 to 0.05)			
	Physical activity				
	Physical activity (Active vs. Inactive)	−0.082 (−0.142 to −0.023)**			
	Regular exercise (1–2 times pw vs. <1 time pw)	−0.16 (−0.23 to −0.089)**			
	Regular exercise (3–4 times pw vs. <1 time pw)	−0.131 (−0.215 to −0.047)**			
	Regular exercise (≥5 times pw vs. <1 time pw)	−0.19 (−0.279 to −0.102)**			
800-m run (min)	BMI			0.023 (0.018,0.027)**	*F* = 42.410
	SES (moderate vs. low)			−0.084 (−0.128,−0.041)**	
	SES (high vs. low)			−0.053 (−0.099,−0.008)*	
	Screen-based SB				
	TV (≥3 h vs. <3 h)			0.149 (0.072,0.227)**	
	C/V use (≥3 h vs. <3 h)			0.03 (−0.047,0.107)	
	Physical activity				
	Physical activity (Active vs. Inactive)			−0.032 (−0.077,0.012)	
	Regular exercise (1–2 times pw vs. <1 time pw)			−0.054 (−0.105,−0.002)*	
	Regular exercise (3–4 times pw vs. <1 time pw)			−0.076 (−0.14,−0.011)*	
	Regular exercise (≥5 times pw vs. <1 time pw)			−0.16 (−0.233,−0.087)**	
Pull-ups (reps)	BMI	−0.164 (−0.209 to −0.118)**	*F* = 15.258 R^2^ = 0.028		
	SES (moderate vs. low)	0.041 (−0.376 to 0.459)			
	SES (high vs. low)	0.595 (0.165 to 1.025)**			
	Screen-based SB				
	TV (≥3 h vs. <3 h)	0.183 (−0.497 to 0.863)			
	C/V use (≥3 h vs. <3 h)	−0.285 (−0.872 to 0.303)			
	Physical activity				
	Physical activity (Active vs. Inactive)	0.466 (0.039, 0.894)*			
	Regular exercise (1–2 times pw vs. <1 time pw)	−0.036 (−0.547 to 0.474)			
	Regular exercise (3–4 times pw vs. <1 time pw)	−0.114 (−0.718 to 0.49)			
	Regular exercise (≥5 times pw vs. <1 time pw)	0.934 (0.294 to 1.574)**			
Bent-leg sit-ups (reps)	BMI			−0.041 (−0.109 to 0.027)	
	SES (moderate vs. low)			1.915 (1.236, 2.594)**	
	SES (high vs. low)			3.204 (2.491, 3.918)**	
	Screen-based SB				
	TV (≥3 h vs. <3 h)			−1.383 (−2.597 to 0.169)*	
	C/V use (≥3 h vs. <3 h)			0.098 (−1.111 to 1.307)	
	Physical activity				
	Physical activity (Active vs. Inactive)			1.07 (0.378, 1.762)**	
	Regular exercise (1–2 times pw vs. <1 time pw)			0.19 (−0.612 to 0.992)	
	Regular exercise (3–4 times pw vs. <1 time pw)			−0.152 (−1.164 to 0.859)	
	Regular exercise (≥5 times pw vs. <1 time pw)			0.651 (−0.49 to 1.792)	

BMI, Body Mass Index; SES, socioeconomic status; Screen-based SB, Screen-based sedentary behavior; TV, Television viewing; C/V use, Computer/videogame use; pw: per week. Data are presented as β coefficient (95% CI). *0.05, **0.01. The model was adjusted for age, BMI, socioeconomic status, physical activity and screen-based sedentary behavior.

## Discussion

This study examined the relationship between BMI, socioeconomic status, sedentary screen behavior, physical activity and physical fitness among Chinese adolescents. We found that all these factors were independently and significantly associated with physical fitness. Adolescents with high levels of physical activity and high socioeconomic status had better physical fitness. Adolescents with obesity and sedentary TV watching behaviors had worse physical fitness. No association was found between computer/video game sedentary behavior and physical fitness.

Regarding frequency of physical activity, previous studies demonstrated that girls generally have lower levels of physical activity than boys (50), and 27.9 % girls were sedentary, compared with 10.6% boys (51). This study found that 36.36% boys and 44.46% girls had low physical activity. The screen behavior of girls may be more severe than boys, so strategies to increase physical activity among adolescents should concentrate more on girls. Previous studies indicate that physical activity is crucial to improving cardiopulmonary endurance, muscle strength and endurance of adolescents (52, 53). The upper limb muscle strength and endurance of boys and the abdominal muscle strength endurance of girls are significantly correlated with physical activity (54), which is consistent with the results shown in this study that boys and girls with high physical activity levels had better upper limb muscle strength and endurance and better abdominal muscle strength and endurance. It is worth noting that the association between physical fitness components and physical activity has been recognized to be gender-specific (55) and that girls are more likely to engage in low to moderate-intensity exercise, while boys are inclined to high-intensity exercise (51). This may be one of the main reasons for this study concluded that boys with high physical activity have better cardiopulmonary endurance and girls with high physical activity have better lower limb explosive power.The World Health Organization (WHO) Guidelines on Exercise and Sedentary Behavior in 2020 suggest that children and adolescents who participate in 60 min or more per day, at least 3 times per week of strenuous aerobic exercise and musculoskeletal exercises, can improve physical fitness (11) and have significant benefits in multiple health and fitness domains and that these benefits persist throughout their lifetime (31). This is consistent with our findings that leisure physical activity five or more times per week was likely to have the greatest impact on physical fitness among adolescents. Therefore, families and schools should enhance intensity and the frequency of leisure physical exercise as one of the pathways for promoting physical fitness in adolescent.

Regarding socio-economic status and BMI, current studies find that physical fitness is related to a wide range of socioeconomic conditions (56). Adolescents with low socioeconomic status may have limited access to the resources and facilities needed to promote physical activity, resulting in less physical activity (57, 58). Physical fitness is positively correlated with regional socio-economic level as the developed districts may provide better educational resources and sport facilities for youth so they have more opportunities to participate in physical activity (57). In the other hand, parents of adolescents in high SES have stronger awareness and ability to supervise their children, resulting in less screen use time (59). However, this study also illustrated the importance of SES in that adolescents with high SES have better lifestyle habits.

The results of this study showed that the adolescents with higher BMI have worse level of physical fitness, which was consistent with previous studies that obesity leads to significant decline in cardiovascular endurance and pulmonary function, speed, strength, flexibility, and other physical qualities (60, 61). In addition, overweight and obesity are the major risk factors for non-communicable diseases that may cause death, musculoskeletal diseases and cancer (62, 63). Obesity may make children watch TV longer (64) and reduce physical activity (65), which will lead to a worse physical fitness. Therefore, government and school departments can improve the physical fitness of adolescents through the prevention or reduction of overweight or obesity.

From the perspective of sedentary behavior on screens, previous studies demonstrated that longer television viewing is associated with the physical fitness of adolescents (66). Watching television is not only positively correlated with metabolic risk factors, but also increases the casual food intake (66, 67) and impacts teens' physical abilities because of the passive nature of TV viewing and lack of social interaction (51). Children who watch TV for more than 2 h per day are more likely to be overweight or obese which implies a dose-response relationship with physical fitness. The more time adolescents spend watching TV each day, the higher the risk of physical fitness decline (24). All these aspects support the finding of this study that adolescents with high TV viewing time have a low level of physical fitness. The present study did not find an association between sedentary behavior (playing computer/video games) and physical fitness in adolescents, which was consistent with the results of previous studies (66). We suggest that some sports-related video game integrating physical activities into real life into the game concept may motive adolescents' interest in physical activities (68). Active video games may prevent weight gain, motivate children for longer periods of physical activity, and improve their healthy lifestyle, thereby improving physical fitness (69). In the future, when formulating strategies to improve the physical fitness of adolescents, we should not only consider screen-based sedentary behaviors, but also formulate more targeted strategies in terms of the classification of screen-based sedentary behaviors.

The strength of this study is that it is the first to use a large sector of the population to analyze the relationship between physical activity, screen-based sedentary behavior and physical fitness for adolescent in China. We hope this study will construct a foundation for future intervention of schools in risk behaviors. The results of this study revealed the current severe state of adolescent physical fitness in China, and provide suggestions for government and schools looking for strategies to improve the physical fitness of Chinese adolescents. However, this study has some limitations that may influence the generalizability of its outcome. First, this study is a cross-section study that cannot accurately explain and analyze the causal relationship. Second, the screen-based sedentary behavior scale jointly considers only the use of computer/video games, but we do not indentify that adolescents can perform different tasks at computer than only playing electronic games, as scholar tasks an social media, which may have a certain impact on our research results. Third, the dietary assessment and sleep behavior was not evaluated so that we can not determine whether those variables are asscoscited with physical activity and physical fitness. Finally, the age stratified physical fitness with respect to BMI, SES and screen based sedentary behavior was not assessed in this study. We will futher explore those issues in the future.

## Conclusions

This study examined the relationship between BMI, socioeconomic status, sedentary screen behavior, physical activity and physical fitness among Chinese adolescents. We found that adolescents with high levels of physical activity and high socioeconomic status were associated with better physical fitness. Adolescents with obesity and sedentary TV watching behaviors were linked to worse physical fitness. Most of these factors were independently and significantly related to physical fitness, but no association was found between computer/video game sedentary behavior and physical fitness. This study suggested that future strategies to improve the physical fitness of Chinese adolescents should focus on adolescents with low socioeconomic status and obesity that promote physical activity and reduce sedentary television-watching behaviors.

## Data Availability Statement

The raw data supporting the conclusions of this article will be made available by the authors, without undue reservation.

## Ethics Statement

The studies involving human participants were reviewed and approved by Ethics Committee of Shandong University (20180517). Written informed consent to participate in this study was provided by the participants' legal guardian/next of kin.

## Author Contributions

XD and XY: funding acquisition and writing—original draft. XD and RZ: methodology. XD, BW, and MD: project administration. XD, XY, RZ, and LD: writing—review and editing. All authors have read and approved the manuscript.

## Funding

This research was funded by the Ministry of Science and Technology of China (2015FY111600).

## Conflict of Interest

The authors declare that the research was conducted in the absence of any commercial or financial relationships that could be construed as a potential conflict of interest.

## Publisher's Note

All claims expressed in this article are solely those of the authors and do not necessarily represent those of their affiliated organizations, or those of the publisher, the editors and the reviewers. Any product that may be evaluated in this article, or claim that may be made by its manufacturer, is not guaranteed or endorsed by the publisher.
